# Reduction of overtreatment without reduction of overdiagnosis in patients with differentiated thyroid cancer: mission impossible

**DOI:** 10.1007/s00423-021-02216-7

**Published:** 2021-06-07

**Authors:** Tjasa Oblak, Andraz Perhavec, Marko Hocevar, Barbara Peric

**Affiliations:** Department of Surgical Oncology, Institute of Oncology Ljubljana, Ljubljana, Slovenia

**Keywords:** Differentiated thyroid cancer, Selective neck dissection, Lymph nodes metastases, Preoperative ultrasound

## Abstract

**Purpose:**

Lateral neck nodal metastases are common in patients with differentiated thyroid cancer (DTC) and usually have an indolent nature. They may be detected via neck palpation or preoperative ultrasound (US) of the neck. We hypothesized that preoperative neck metastases detected with US did not affect regional recurrence or long-term survival.

**Methods:**

A retrospective analysis of patients’ records treated for DTC at our institution between January 2006 and December 2016 was performed. Information about preoperative US of the neck, treatment, demographics, staging, and histopathology was obtained. The endpoints for the study were nodal recurrence and survival. Differences in survival were analyzed between three groups of patients divided by presence or lack of preoperative US and/or palpable cervical lymph nodes (PLN). Furthermore, the prognostic value of multiple variables was tested by univariate and multivariate analysis.

**Results:**

There were 1108 patients with DTC, 221 males and 887 females. The median age was 48.3 years (range 3 to 86), the median time of observation was 68 months (range 0 to 142). Eight hundred sixty-two patients without PLN or preoperative US represented group 1, 112 patients with PLN were in group 2, and 134 patients without PLN and with preoperative US were in group 3. Only five patients had a regional recurrence, one died due to distant metastases. There was no statistically significant difference in survival between the groups (p = 0.841) and neck US was not significantly associated with overall survival neither in univariate nor in multivariate analysis.

**Conclusion:**

In patients with DTC, the benefits of preoperative US of cervical lymph nodes are probably limited and “less is more” approach is advised.

## Introduction

Differentiated thyroid cancer (DTC) is one of the most common endocrine cancers and its incidence in the developed countries has increased steeply over the last decades [1]. On the contrary, the number of deaths due to DTC did not increase proportionally or has even declined [2, 3]. Major rise in DTC incidence is largely attributed to overdiagnostics due to the increasing availability of different imaging tools, mostly ultrasound (US), CT, and PET-CT, that allowed detection of otherwise undetectably small or indolent neoplasms [3, 4].

The same imaging modalities are used to detect possible lymph node metastases. Lateral neck lymph node metastases are present in 20–60% of patients with DTC [5–7]. Due to the characteristically indolent nature of such metastases, the involvement of regional lymph nodes has a negligible prognostic value in younger patients, while it affects the staging and survival outcome in patients above the age of 55 [8, 9]. While clinically occult lymph node metastases have little prognostic value, macroscopic metastatic lymph nodes increase the risk of both local and distant recurrence in patients older than 55 years. Lateral neck lymph node metastases that are greater than 3 cm in size lead to a decrease in recurrence-free survival and in disease-specific survival [10, 11].

While we are witnessing de-escalating treatment of primary tumors with lobectomy instead of total thyroidectomy in patients with DTC or even active surveillance instead of surgery in papillary microcarcinomas, this is not the case with lymph node metastases [11, 12]. The ATA guidelines state as follows: “Preoperative neck US for cervical (central and especially lateral neck compartments) lymph nodes is recommended for all patients undergoing thyroidectomy for malignant or suspicious for malignancy cytological or molecular findings. US-guided FNA of sonographically suspicious lymph nodes ≥8–10 mm in the smallest diameter should be performed to confirm malignancy if this would change management. Detection of a single abnormal cervical lymph node is considered an indication to perform a compartmental lymph node dissection” [13].

Not all thyroid surgeons would agree to such treatment. Therefore, a more conservative approach to regional lymph nodes is proposed by Dutch national guidelines which state: “While it is possible with thyroid cancer to detect small neck node metastases using ultrasound and ultrasound-guided aspiration cytology, the preoperative benefit of this is limited because neck node metastases are only of marginal prognostic significance.” The same guidelines also state that for small (<1 cm) thyroid carcinoma neck lymph node metastases that have been detected preoperatively, a wait-and-see approach may be considered and possible neck node dissection for residual disease may be performed after treatment of the primary tumor [14, 15]. Despite a more conservative approach which results in higher percentages of bigger primary tumors and lower percentage of lymph nodal metastases in Dutch population, their 10-year overall survival is excellent and comparable to survival in USA population [3].

Our study was conceived assuming that preoperative US of cervical lymph nodes may represent just another example of overdiagnostics and overtreatment in patients with DTC.

The primary endpoint of our study was to evaluate if preoperative US of cervical lymph nodes can statistically significantly affect long-term recurrence and overall survival in patients with DTC that had been treated at our institution.

The secondary endpoints were to assess whether other patients’ DTC characteristics may be associated with statistically significant differences in survival and structural relapse of the disease.

## Materials and methods

A retrospective analysis of 2231 patients with thyroid surgery performed between January 2006 and December 2016 at the Institute of Oncology Ljubljana, Slovenia, was performed. Final histopathological report confirmed thyroid malignancy in 1233 patients. The inclusion criteria were as follows: patients with differentiated thyroid cancer (papillary carcinoma – classic and variants, follicular carcinoma, and Hürthle cell carcinoma) treated at our center in the years 2006–2016. Patients with medullary or anaplastic thyroid carcinoma as well as other rare thyroid malignancies were excluded. Therefore, only 1108 patients with DTC were selected for further analysis.

**Fig. 1:**
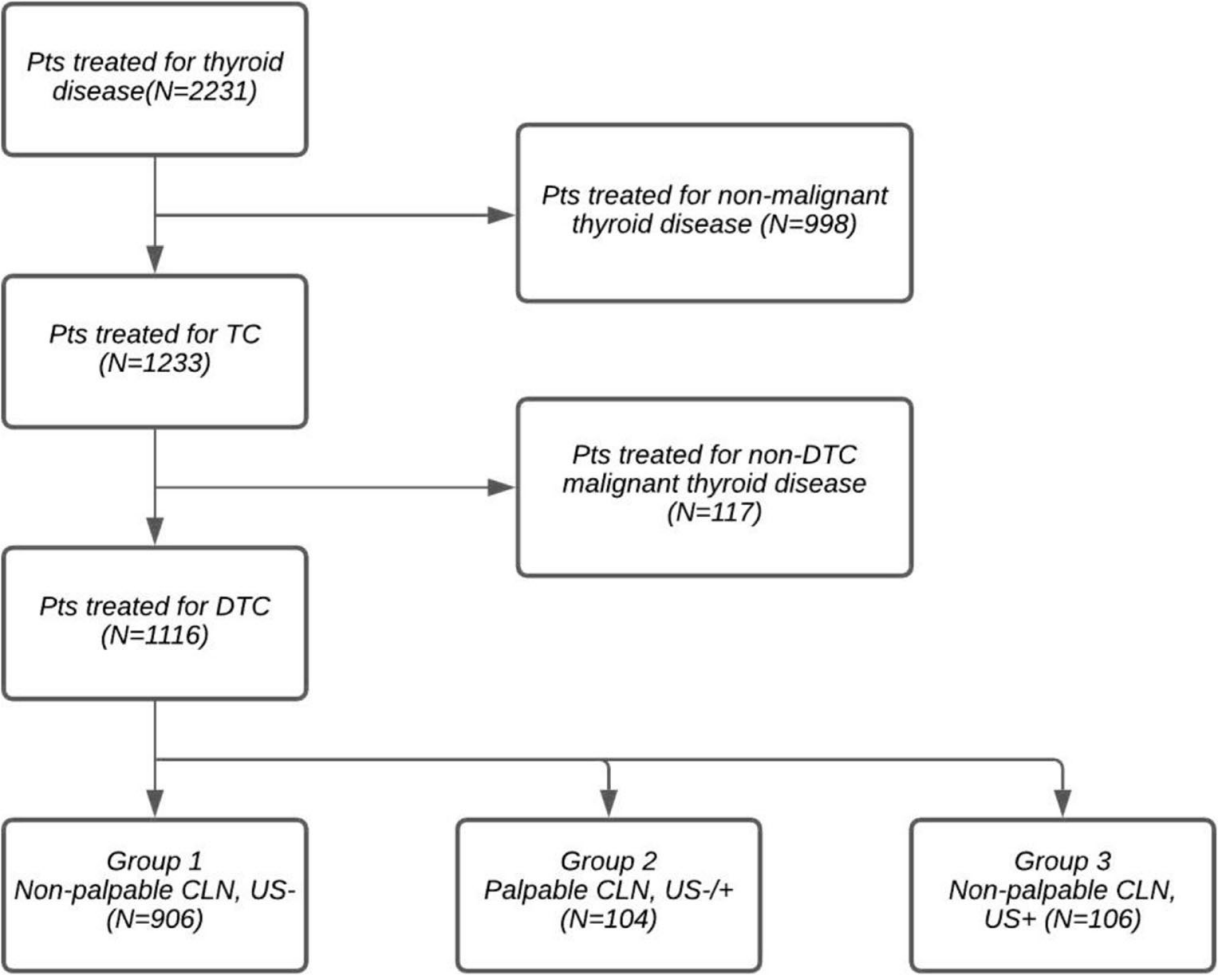
Flow of patients in the study.* Pts *patients, *TC* thyroid cancer, *DTC *differentiated thyroid cancer, *CLN *cervical lymph nodes,* US+ *neck ultrasound performed, *US- * neck ultrasound not performed

Patients were divided into three groups based on palpability of cervical lymph nodes and whether preoperative neck US was performed. Flow of patients is presented in Fig. 1.

Palpation of lateral neck lymph nodes was performed in all patients at the first clinical visit, while preoperative neck US for cervical lymph nodes was performed only in selected cases. The decision on performing preoperative neck US was mainly based on the preoperative cytological result of the thyroid nodule. Namely, only 2.6% of patients with Bethesda I–IV nodules underwent preoperative neck US. On the contrary, neck US was performed in 29.3% patients with Bethesda V and 44.9% patients with Bethesda VI thyroid nodules. The next most important factor was the surgeons’ preference—some surgeons at the institute decided to perform preoperative neck US more often than others.

Preoperative US of cervical lymph nodes was performed by experienced radiologists dedicated to neck pathology at the institute. The boundaries of the neck lateral compartment were defined as described by the American Joint Committee on Cancer (AJCC). The presence of the following was defined as sonographic abnormalities: enlarged size, loss of a fatty hilum, a round shape, taller than wide shape, hyperechogenicity, cystic change, calcifications, and peripheral vascularity. In all patients with sonographically abnormal lymph nodes, US-guided FNA was performed. If metastases to the cervical lymph nodes were confirmed, a therapeutic neck dissection was performed at the time of thyroidectomy encompassing neck levels II to V.

When the final histopathology report was obtained, all patients were staged according to the latest 8^th^ edition AJCC system.

Adjuvant radioiodine ablation with 100 mCi was performed in all patients with tumors bigger than 1 cm and/or N1/2 disease at the time of thyroid hormone withdrawal or rhTSH stimulation.

After treatment, all patients were included in regular follow-up at our outpatient clinic with clinical assessment of the neck, TSH, fT3, fT4, and thyroglobulin (Tg) and anti-Tg antibodies measurements every 6 months during the first 2 years and yearly thereafter. In the case of elevated Tg (> 2 ng/ml) or clinical suspicion of disease recurrence, an US assessment of the neck was performed with US-guided FNA of suspicious lymph nodes size ≥ 8 mm.

The time to recurrence and overall survival were measured in months from the date of surgery to the date when recurrence or death was detected.

IBM SPSS Statistics 21 was used to generate data analysis. Continuous patient characteristics were summarized as medians and ranges; categorical characteristics were summarized as proportions and percentages. Three groups of patients were formed based on clinical and sonographic assessment of the neck for the purpose of survival analysis (Table 1). The cumulative overall survival time was calculated by the Kaplan-Meier method and analyzed by the log-rank test. The prognostic value of multiple variables was tested by univariate and multivariate Cox regression analysis. P < 0.05 was considered statistically significant.

**Table 1:** Groups for survival analysis

	Palpable lymph nodes	US of cervical lymph nodes
Group 1	No	No
Group 2	Yes	Yes or No
Group 3	No	Yes

The study was approved by the Institutional Ethics Committee.

## Results

The clinicopathological characteristics of the study group are summarized in Table 2. There were 221 males and 887 females with a median age of 48.3 years (range 3 to 86). The median time of observation was 68 months (range 0 to 142).

**Table 2:** The clinicopathological characteristics of the cohort. *TT*, total thyroidectomy; *CND*, central neck dissection; *LND*, lateral neck dissection

Variable	No. of patients	%
Sex		
Female	887	80.1%
Male	221	19.9%
Age		
Median: 48.3 y (range 3–86)		
Younger than 55	682	61.6%
Older than 55	426	38.4%
Cytopathology (Bethesda)		
I	40	3.6%
II	40	3.6%
III	25	2.3%
IV	246	22.2%
V	53	4.8%
VI	704	63.5%
Histopathology		
Papillary cancer	1038	93.7%
Follicular cancer	70	6.3%
T stage		
T1	627	56.6%
T2	260	23.5%
T3	210	19.0%
T4	11	1.0%
N		
N1/2	252	22.7%
N0	856	77.3%
M		
Yes	6	0.5%
No	1102	99.5%
Extrathyroideal extension		
No	836	75.4%
Yes	217	19.6%
Minimal	55	5%
Type of surgery		
Total thyroidectomy	986	
- Only TT	- 722	65.2%
- TT and CND	- 120	10.8%
- TT, CND, and LND	- 136	12.3%
Lobectomy	130	11.7%
Adjuvant radioiodine ablation		
Yes	656	59.2%
No	452	40.8%

A first group comprised of 862/1108 (77.8%) patients with non-palpable lymph nodes and no preoperative neck US of whom 123 (14.3%) had a lobectomy, 668 (77.5%) had a TT, and 71 (8.2%) had TT and CND.

A second group comprised of 112/1108 (10.1%) patients with palpable lymph nodes and FNA was performed in all of them. FNA was US-guided in 109 (97.3%) patients. In 73 (65.1%) patients, FNA confirmed lymph node metastases, in 27 (24.1%) cases FNA was negative, and while in 12 (10.7%) patients FNA was non-diagnostic. Eighty-eight (78.6%) patients had TT + CND + LND: all patients with confirmed cervical metastases and non-diagnostic FNA, plus 3 patients with negative FNA but with US highly suspicious for metastatic involvement. Of the remaining 24 (21.4%) patients, 11 (9.8%) had TT and CND and 13 (11.6%) had only TT. In the latter group, 3 patients had highly suspicious US for lymph node metastases with negative FNA and were treated with TT and selected enlarged lymph nodes removal because of several important comorbidities.

A third group comprised of 134/1108 (12.1%) patients with non-palpable lymph nodes and preoperative neck US for cervical lymph nodes. US-guided FNA confirmed lateral neck lymph node metastases in 36 (26.9%) patients, FNA was non-diagnostic in 12 (9.0%), and negative in 86 (64.2%) cases. In 48 (35.8%) patients, TT, CND, and LND was performed, i.e., in all patients with positive or non-diagnostic FNA. Of the remaining 86 (64.2%) patients with negative preoperative US, 38 (28.4%) had TT and CND and 48 (35.8%) had TT.

A total of 99 patients with clinical N0 had a pathologic N1 disease.

Locoregional disease relapse was detected during follow-up in 6/1108 (0.5%) patients, and the mean time to recurrence was 32 months (range 7 to 60 months). Characteristics of those patients are summarized in Table 3.

**Table 3:** Characteristics of patients with locoregional relapse. *PLN*, palpable lymph nodes; *FNA*, fine needle aspiration; *US*, ultrasound; *TTR*, time to recurrence

	Patient 1	Patient 2	Patient 3	Patient 4	Patient 5	Patient 6
Age	26	34	58	40	31	29
PLN	No	No	No	No	No	No
Neck US	Yes	No	No	No	Yes	No
FNA	Non-diagnostic	No	No	No	No	No
T stage	2	1	1	3	3	3
Primary surgery	TT+CND	TT+CND	TT+CND	TT	TT	TT
TTR (months)	24	18	24	7	60	60

Only twelve patients were lost to follow-up, four patients shortly after surgery (due to change of residency or patients from outside the country), and the other patients were lost at respectively 12, 20, 24 (two patients), 33, 36, 58, and 86 months of follow-up.

**Fig. 2:**
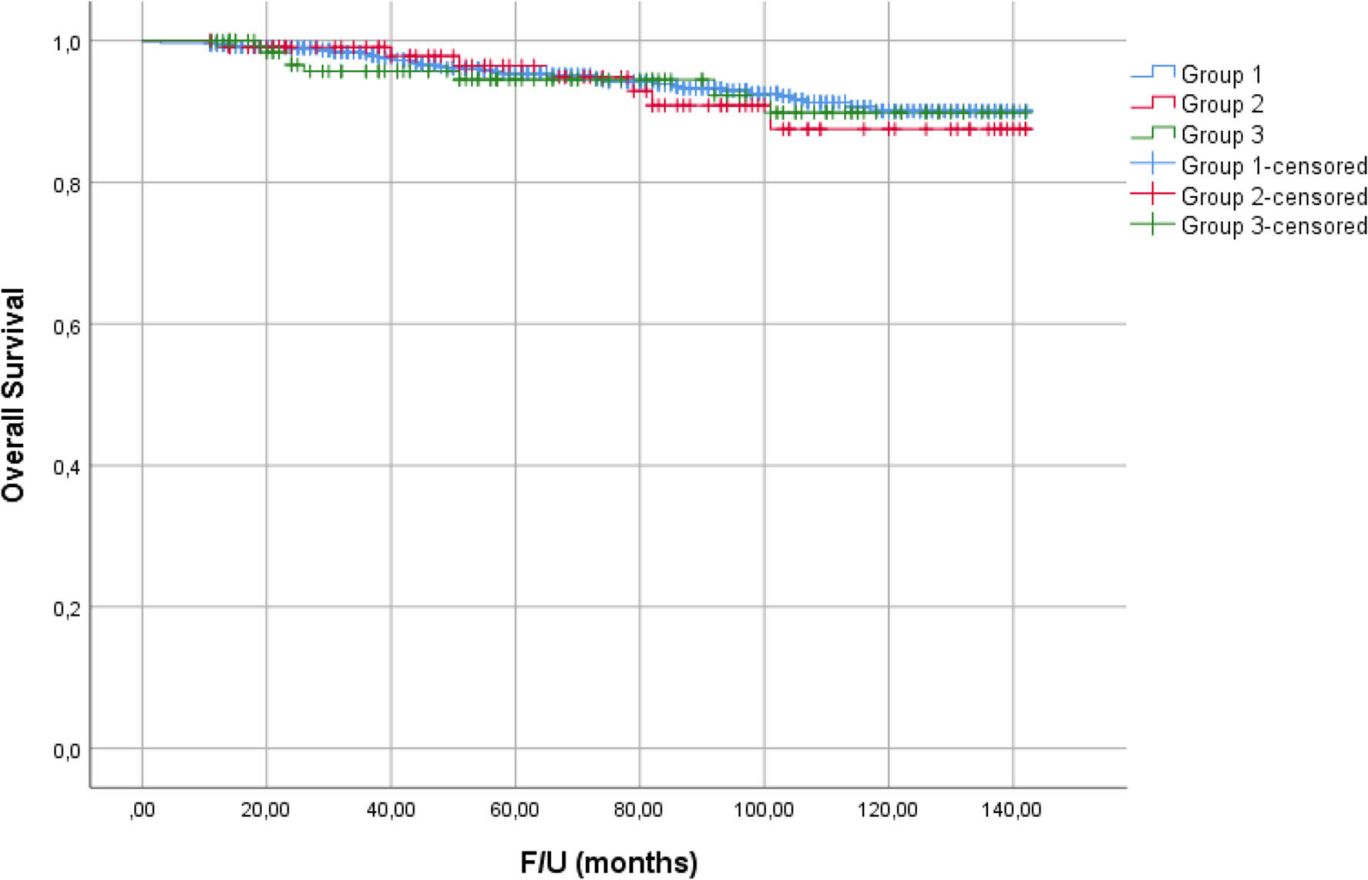
Survival curves for three groups of patients. Group 1: patients without palpable cervical lymph nodes or preoperative US; group 2: patients with palpable cervical lymph nodes with or without preoperative US; group 3: patients without palpable cervical lymph nodes and with preoperative US

There was no statistically significant difference in survival between the three groups (p = 0.841). Estimated 10-year survival rate for group 1, 2, and 3 was 90.3%, 87.5%, and 89.9%, respectively (Fig. 2).

Statistically significant differences in age as well as T stage, performance of adjuvant radioiodine ablation, and number and diameter of metastatic lymph nodes between the three groups were observed (Table 4). There were no statistically significant differences in gender, histopathology, N and M stage, palpable lymph nodes, performance of preoperative neck US, and positive FNA.

**Table 4:** Differences in age, T stage, and number and diameter of metastatic lymph nodes between the three groups of patients. *MLN*, metastatic lymph nodes; *RIA*, radioiodine ablation

	Group 1 (n = 862)	Group 2 (n = 112)	Group 3 (n = 134)	p-value
Age (years)	49.5	43.9	44.4	0.002
T stage				0.020
- T1	505 (58.6%)	61 (54.5%)	61 (45.5%)	
- T2	210 (24.4%)	11 (9.8%)	39 (29.1%)	
- T3	138 (16.0%)	39 (34.8%)	33 (24.6%)	
- T4	9 (1.0%)	1 (0.9%)	1 (0.7%)	
Mean no. of MLN	0.18	9.46	3.94	< 0.001
Mean diameter of MLN (mm)	0.2	20.4	8.4	< 0.001
Adjuvant RIA				0.002
- Yes	487 (56.5%)	79 (70.5%)	90 (67.2%)	
- No	375 (43.5%)	33 (29.5%)	44 (32.8%)	

In univariate overall survival analysis, gender, age, and histopathology T stage and M stage were statistically significant. Multivariate analysis that included all clinically relevant variables confirmed only age and histopathology to be significantly associated with overall survival (Table 5).

**Table 5:** Results of multivariate analysis. *MLN*, metastatic lymph nodes; *PLN*, palpable lymph nodes; *US*, ultrasound; *FNA*, fine needle aspiration; *RIA*, radioiodine ablation; *HR*, hazard ratio

	HR	95.0% CI for HR		p
		Lower	Upper	
Gender (male vs. female)	.628	.175	2.225	.476
Histopathology (follicular vs. papillary)	.122	.019	.804	.029
Age	1.093	1.047	1.140	.000
T stage	.899	.456	1.772	.759
N	.238	.036	1.586	.138
M stage	9.467	.819	109.407	.072
PLN (palpable vs. non-palpable)	.915	.198	4.235	.910
US (performed vs. not performed)	.872	.268	2.838	.820
No. of MLN	1.015	.917	1.123	.773
Size of MLN	1.026	.982	1.072	.245
FNA result (positive vs. negative) RIA	1.774 0.993	.649 0.513	4.845 1.923	.264 .983

## Discussion

DTC in general has one of the highest cure rates among all malignant diseases with 10-year survival exceeding 90–95% [7]. Moreover, it is unique among epithelial cancers because regional lymph node metastases are present in very high percentage but affect survival only in patients older than 55 years. With the increasing usage of the US, DTC became one of the most overdiagnosed and overtreated cancers.

Results of our study confirmed that DTC in Slovenia has an excellent survival rate (10-year survival of 90.3%) that is comparable to data from other countries worldwide [16]. Furthermore, according to our results preoperative ultrasound of cervical lymph nodes has no prognostic value for long-term recurrence and survival neither has surgical treatment of neck metastases found by preoperative diagnostics. In addition, locoregional recurrences were observed in only six patients (0.5%) and can hardly be associated with any specific risk factor, including preoperative US. The observed really low percentage may be the result of DTC (over)treatment at the time: until 2016 all Slovenian patients with DTC > 1 cm had total thyroidectomy and postoperative adjuvant radioiodine ablation with 100 mCi with thyroid hormone withdrawal or rhTSH stimulation. Deescalation of treatment followed in 2017 when a revision of the national guidelines was made.

The main question when dealing with a disease with such an excellent prognosis is the unnecessary morbidity as a result of overdiagnosis and overtreatment. As CND and LND are procedures with a higher percentage of hypoparathyroidism and nerve injury they should be avoided whenever possible. One possible way to avoid unnecessary surgery could be omitting preoperative US of cervical lymph nodes. According to the results of our study, clinical evaluation of cervical lymph nodes without US enables survival similar to the group with preoperative US with low rates of unexpected locoregional recurrences. Clinical evaluation can, on the other hand, spare the patient from unnecessary lymph node dissection. In 48 patients in our study, LND was performed only because of a positive result of preoperative neck US. We are aware that these results are in contrast with other studies underlying the importance of preoperative neck US and even suggesting that more accurate and extensive preoperative neck diagnostic are needed in order to reduce the chances of relapse [17–19]. A possible explanation of such discrepancies may lie in population characteristics. Most of our patients had small tumors with favorable prognostic factors. Few patients had extrathyroideal extension of the tumor and most of the metastases in the cervical lymph nodes were small-sized (less than 3 cm)—both characteristics are not associated with a decreased survival rate. Furthermore, nearly half of our patients were younger than 45 years and the involvement of regional lymph nodes has a negligible prognostic value in younger patients [7, 8].

Once the patient is informed about the US detected and by FNA confirmed lymph node metastasis, the decision to omit lymph node dissection is very difficult and may lead to unnecessary patients’ stress. It is important to use preoperative US of cervical lymph nodes less often and more selectively. A reasonable compromise would be to indicate preoperative US of cervical lymph nodes only in patients older than 55 years where lateral neck lymph node metastases lead to a decrease in recurrence-free survival and in disease-specific survival.

Another important aspect of overtreatment because of overdiagnostics in patients with DTC is additional use of adjuvant radioiodine ablation. Patients with US detected lymph node metastases as a rule have lymph node metastases bigger than 2 mm and as such belong to an ATA intermediate-risk level where radioiodine adjuvant therapy should be considered after total thyroidectomy according to the recommendation 51 in ATA guidelines [13, 20–22]. The median size of lymph node metastases detected by preoperative US in group 3 was 8.4 mm in our patients and they were all treated with adjuvant radioiodine ablation in addition to lymph node dissection which might further contribute to overtreatment.

Retrospective analysis is the main weakness of our study. Nevertheless, since our institution is the only thyroid cancer center in the country, where all patients with thyroid cancer are directed, the results are clearly representative for the whole Slovenian population. Follow-up is also performed at our institution, therefore minimizing attrition bias.

## Conclusions

In patients with DTC, the benefits of preoperative US of cervical lymph nodes are probably limited. In order to avoid overtreatment in the form of lymph node dissection and adjuvant radioiodine ablation, we advise a “less is more” approach.

## References

[1] Ervik M, Lam F, Ferlay J, et al (2016) Cancer today. Lyon, France: International Agency for Research on Cancer. Cancer Today. Available from: http://gco.iarc.fr/today. Accessed 14 Jul 2018

[2] Lim H, Devesa SS, Sosa JA, Check D, Kitahara CM (2017). Trends in thyroid cancer incidence and mortality in the United States, 1974-2013. JAMA..

[3] La Vecchia C, Malvezzi M, Bosetti C (2015). Thyroid cancer mortality and incidence: a global overview. Int J Cancer.

[4] Ahn HS, Kim HJ, Welch HG (2014). Korea’s thyroid-cancer “epidemic” – screening and overdiagnosis. N Engl J Med.

[5] Roh JL, Kim JM (2011). Park CI Ann Central lymph node metastasis of unilateral papillary thyroid carcinoma: patterns and factors predictive of nodal metastasis, morbidity, and recurrence. Surg Oncol.

[6] Pereira JA, Jimeno J, Miquel J (2005). Nodal yield, morbidity, and recurrence after central neck dissection for papillary thyroid carcinoma. A Surgery.

[7] Gharib H, Papini E, Garber JR, et al (2016) American Association of Clinical Endocrinologists, American College of Endocrinology, and Associazione Medici Endocrinologi Medical Guidelines for Clinical Practice for the Diagnosis and Management of Thyroid Nodules – 2016 Update. Endocrine Practice, Vol. 22, No. Supplement 1, pp 1-60 10.4158/EP161208.GL 27167915

[8] Nixon IJ, Wang LY, Migliacci JC, Eskander A, Campbell MJ, Aniss A, Morris L, Vaisman F, Corbo R, Momesso D, Vaisman M, Carvalho A, Learoyd D, Leslie WD, Nason RW, Kuk D, Wreesmann V, Morris L, Palmer FL, Ganly I, Patel SG, Singh B, Tuttle RM, Shaha AR, Gönen M, Pathak KA, Shen WT, Sywak M, Kowalski L, Freeman J, Perrier N, Shah JP (2016). An international multi-institutional validation of age 55 years as a cutoff for risk stratification in the AJCC/UICC staging system for well-differentiated thyroid cancer. Thyroid..

[9] Nixon IJ, Kuk D, Wreesmann V, Morris L, Palmer FL, Ganly I, Patel SG, Singh B, Tuttle RM, Shaha AR, Gönen M, Shah JP (2016). Defining a valid age cutoff in staging of well-differentiated thyroid cancer. Ann Surg Oncol.

[10] Ito Y, Fukushima M, Tomoda C (2009). Prognosis of patients with papillary thyroid carcinoma having clinically apparent metastasis to the lateral compartment. Endocr J.

[11] Sugitani I, Kasai N, Fujimoto Y, Yanagisawa A (2004). A novel classification system for patients with PTC: addition of the new variables of large (3 cm or greater) nodal metastases and reclassification during the follow-up period. Surgery.

[12] Leboulleux S, Tuttle RM, Pacini F, Schlumberger M (2016). Papillary thyroid microcarcinoma: time to shift from surgery to active surveillance?. Lancet Diabetes Endocrinol.

[13] Haugen BR, Alexander EK, Bible KC, Doherty GM, Mandel SJ, Nikiforov YE, Pacini F, Randolph GW, Sawka AM, Schlumberger M, Schuff KG, Sherman SI, Sosa JA, Steward DL, Tuttle RM, Wartofsky L (2016). 2015 American Thyroid Association management guidelines for adult patients with thyroid nodules and differentiated thyroid cancer: the American Thyroid Association guidelines task force on thyroid nodules and differentiated thyroid cancer. Thyroid..

[14] Werkgroepleden. Richtlijn Schildkliercarcinoom (2015) (Guideline: thyroid cancer including diagnostics of the nodule). 2.0; available online: https://www.oncoline.nl/index.php?pagina=/richtlijn/item/pagina.php&id = 38754&richtlijn_id = 978. Accessed April 2020

[15] Metman MJH, Lončar I, Kruijff S (2020). Is less always more in a national well-differentiated thyroid cancer population?. Eur J Surg Oncol.

[16] Siegel RL, Miller KD, Jemal A (2017). Cancer statistics (2017). CA Cancer J Clin.

[17] Bongers PJ, Verzijl R, Dzingala M, Vriens MR, Yu E, Pasternak JD, Rotstein LE (2019). Preoperative computed tomography changes surgical management for clinically low-risk well-differentiated thyroid cancer. Ann Surg Oncol.

[18] Lin MC, Yin L (2019). Value of dual-energy computed tomography for diagnosing cervical lymph node metastasis in patients with papillary thyroid cancer. J Comput Assist Tomogr.

[19] Cho SJ, Suh CH, Baek JH, Chung SR, Choi YJ, Lee JH (2019). Diagnostic performance of CT in detection of metastatic cervical lymph nodes in patients with thyroid cancer: a systematic review and meta-analysis. Eur Radiol.

[20] Grebe SK, Hay ID (1996). Thyroid cancer nodal metastases: biologic significance and therapeutic considerations. Surg Oncol Clin N Am.

[21] Scheumann GF, Gimm O, Wegener G, Hundeshagen H, Dralle H (1994). Prognostic significance and surgical management of locoregional lymph node metastases in papillary thyroid cancer. World J Surg.

[22] Qubain SW, Nakano S, Baba M, Takao S, Aikou T (2002). Distribution of lymph node micrometastasis in pN0 well-differentiated thyroid carcinoma. Surgery..

